# Ipsilateral Femoral Fracture Non-Union and Delayed Union Treated By Hybrid Plate Nail Fixation and Vascularized Fibula Bone Grafting: A Case Report

**DOI:** 10.5704/MOJ.1307.015

**Published:** 2013-07

**Authors:** CK Chan, WM Ng, AM Merican

**Affiliations:** Department of Orthopaedic Surgery, University of Malaya, Kuala Lumpur, Malaysia; Department of Orthopaedic Surgery, University of Malaya, Kuala Lumpur, Malaysia; Department of Orthopaedic Surgery, University of Malaya, Kuala Lumpur, Malaysia

## Abstract

Non-union is a well recognized complication of femoral neck fractures. The decision whether to attempt fracture fixation or to resort to hip replacement is particularly difficult in patients in the borderline age group in whom complex attempts at gaining union may fail and later present a difficult revision. On the other hand the patient may be young enough that arthroplasty best be avoided . Besides, presence of ipsilateral femoral shaft fracture with delayed union in addition to the femoral neck non-union will pose major problems at operation. We share our experience in treating a femoral neck fracture non-union with ipsilateral femoral shaft delayed union in the shaft and in the distal femur in a fifty years old patient. The fracture was treated with an angle blade plate and supracondylar nail supplemented with a free vascularised fibular bone grafting and autologous cancellous graft. There was radiological union at fourth month. At sixth months, the patient was free of pain and able to walk without support. Thus, we would like to suggest that vascularised fibula bone grafting with supracondylar nailing is a viable option for this pattern of fracture.

**Key Words:**

Vascularized fibular bone graft, neck of femur, femoral shaft fracture, non-union

## Introduction

Fracture neck of femur is well known for complications of non-union and osteonecrosis of the head, up to 40%^1^. In order to treat non-union of the femoral neck, the principles and techniques that can be used are important. Many techniques have been addressed for the treatment of femoral neck non-union, such as rigid internal fixation with or without bone grafting, muscle pedicle bone graft, valgus osteotomy of the proximal femur with or without bone graft, or hip arthroplasty^2^.

We report a case with neglected femoral neck fracture with neck non-union and ipsilateral delayed unions of fractures at the midshaft and supracondylar region. This was treated with vascularized fibular bone graft together with supracondylar nailing.

## Case Report

**Fig. 1:**
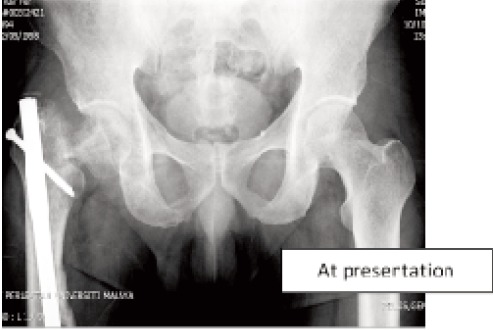
: Shows there was fracture neck of right femur extending to subcapital region of the neck after five months from initial injury. The fracture was intracapsular and the head was in varus position.

**Fig. 2a & 2b:**
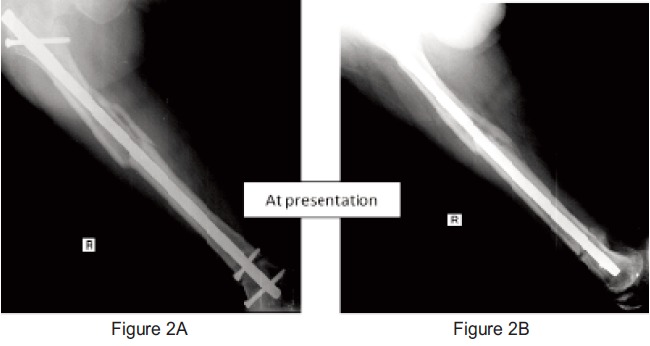
: Show presence of non-union of the supracondylar region of right femur and delayed union of the midshaft of right femur.

A 50-year-old man sustained a closed mid-shaft and supracondylar fractures of the right femur in a motor vehicle accident in May 2008. He was treated with an interlocking nail in a private hospital. Postoperative radiographs revealed a crack in the femoral neck. Subsequently, the femoral neck fracture displaced. He was seen in our institution in November 2008 with an antalgic and short limb gait. There was dull pain in the right groin and there was 2.5 centimetres shortening. His Harris hip score was 55%. Radiographs revealed non-union of the femoral neck and delayed union of the midshaft as well as at the supracondylar region ( Figure 1 & Figure 2).

Bone scan confirmed femoral head viability. After a detailed discussion regarding treatment options, postoperative rehabilitation, risks and complications, he consented for surgery: vascularized fibula bone grafting, angle blade plating, supracondylar nailing and cancellous bone grafting.

SURGICAL TECHNIQUESPatient was positioned on semi-lateral position on a radiolucent table without traction. The whole lower limb was draped free. The interlocking nail was removed.

An anterolateral (Watson Jones) approach was used to expose the hip. The ascending branch of the lateral circumflex femoral vessels was identified. The femoral neck fracture was reduced and provisionally stabilized. Under fluoroscopy, an angle blade plate was inserted inferiorly in the femoral neck and head. A dynamic hip screw triple reamer was used to create a core through the lateral cortex of the proximal femur into the femoral head. A vascularized fibular graft was harvested from the ipsilateral lower limb and trimmed to the appropriate length. At the same time, cancellous bone graft was harvested from the ipsilateral iliac crest.

**Fig. 3:**
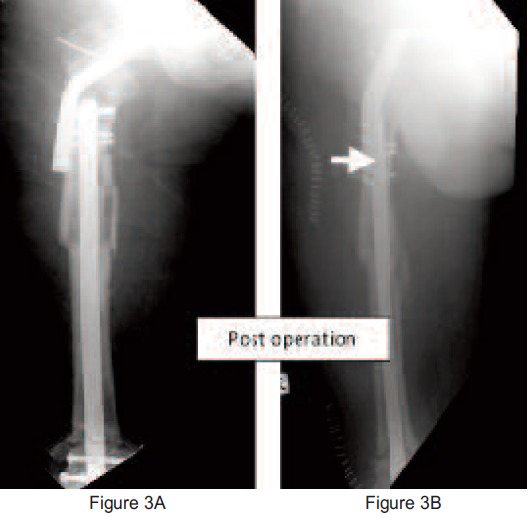
: Shows that the fracture was reduced well and the fractures were fixed by supracondylar nail and angle blade plate. The vascularized fibula bone graft was stabilized by a Kirshner wire. Varus deformity of the neck was corrected. The white arrow shows the screw was incorporated into the angle blade plate.

The core was packed with cancellous bone graft leaving an appropriate space for the fibula graft and its vascular pedicle. The fibular bone graft was stabilized with a Kirshner wire and anastomosis was performed under loupes magnification. The femoral shaft fracture and supracondylar fracture were fixed with a supracondylar nail and the distal fracture was bone grafted. The proximal locking screw was incorporated in the angled blade plate. ( Figure 3)

Postoperatively, he was advised strictly non weight bearing for eight weeks followed by partial weight bearing for another four weeks. Full weight bearing was commenced three months postoperatively.

**Fig. 4:**
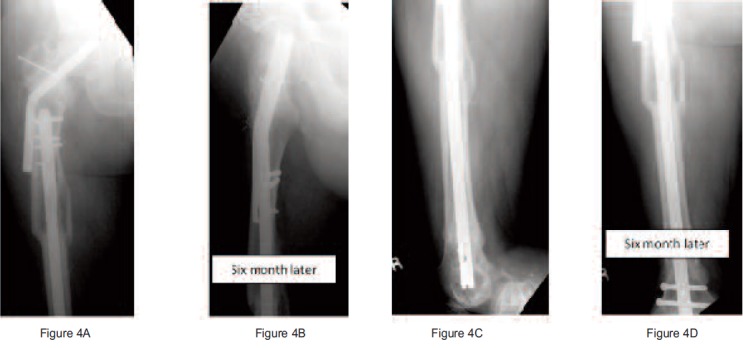
: Shows union of the right femoral neck after six months. There was no osteonecrosis of the femoral head. Shows union of the right femoral shaft after six months.

Radiograph at sixth months postoperative revealed union of the femoral neck and shaft of the femur. (Figure 4) He was able to walk and climb stairs without support and he had no pain. The right hip motion was equal to the normal side but there was 1.5 cm of shortening. Harris Hip score was 94% post operatively. Bone scan was performed at this time revealed that the head was viable.

## Discussion

The management for this non-union of the femoral neck was difficult due to the young age of the patient and complexity of the fracture. Many surgeons would think that prosthetic replacement of the hip is the only alternative. However, the long term results of hip arthroplasties, especially in younger people are not always as good as expected 3. Additionally, in this patient, the ipsilateral non-union and delayed union would complicate arthroplasty.

Our main aim in managing this patient was to attain union of fracture and to preserve the vascularity of femoral head. We did not perform valgisation osteotomy, which is suggested by many surgeons because the Pauwel’s angle of this femoral neck fracture was more than 75°, which required a lot of bone needed to be resected. Besides, valgisation osteotomy might carry the risk of avascular necrosis of the head of femur if overcorrected^4^. Furthermore, the presence of femoral shaft fracture also increased the difficulties of osteotomy in this patient.

We chose the vascularised fibular bone grafting based on its numerous advantages, such as its ability to maintain its osseus structure, its active participation in the bone healing process, hypertrophy in response to load demand, strong cortical bone with straight shape, and simple harvesting technique^5^. Additionally, the vascularity provided would be advantageous should the surgical insult itself cause osteonecrosis.

Angle blade plate was chosen in this case as it was the best implant that could be used to stabilize the femoral head and thus offered stability to allow the live fibula graft to incorporate with surrounding bone. Iliac cancellous bone graft was used as well to enhance the healing process of the fracture.

The outcome of this treatment in this patient was encouraging as the fracture attained union; the patient regained excellent function and range of motion of the hip with no radiological evidence of femoral head osteonecrosis.

In conclusion, vascularised fibular bone grafting with angle blade plate is a viable alternative for treatment of non-union fracture of femoral neck, particularly in young. Besides, combination with supracondylar nailing also may be a viable option for this difficult fracture management.

## References

[1] van Vugt Arie B. (2007). Femoral neck non-unions: How do l do it?. Injury Int J Care Injured.

[2] Canale ST, Campbell WC (1998). Campbell’s Operative Orthopaedics. 9th ed.

[3] Nagi ON, Dhillon MS (2003). Management of neglected/un-united fractures of the femoral neck in young adults. Current Orthopaedics.

[4] Said GZ, Farouk O, Said HGZ (2010). Valgus intertrochanteric osteotomy with single-angled 130º plate fixation for fractures and non
union of the femoral neck. Int Orthop.

[5] Sowa DT, Weiland AJ (1987). Clinical applications of vascularised bone autografts. Orthop Clin North Am.

[6] LeCroy CM, Rizzo M, Gunneson E (2002). Urbaniak JR. Free vascularized fibular bone grafting in the management of femoral neck
non-union in patients younger than fifty years. J Orthop Trauma.

